# The Influence of Non-Pharmacological and Pharmacological Interventions on the Course of Autosomal Dominant Polycystic Kidney Disease

**DOI:** 10.3390/nu16183216

**Published:** 2024-09-23

**Authors:** Karolina Kędzierska-Kapuza, Inga Łopuszyńska, Grzegorz Niewiński, Edward Franek, Małgorzata Szczuko

**Affiliations:** 1 Department of Internal Diseases, Endocrinology and Diabetology, National Medical Institute of the Ministry of Interior and Administration, 137 Wołoska St., 02-507 Warsaw, Poland; edward.franek@cskmswia.gov.pl; 2 Department of Gastroenterological Surgery and Transplantology, National Medical Institute of the Ministry of Interior and Administration, 137 Wołoska St., 02-507 Warsaw, Poland; ingalopuszynska@gmail.com (I.Ł.); grzegorz.niewinski@cskmswia.gov.pl (G.N.); 3 Department of Human Nutrition and Metabolomic, Pomeranian Medical University, 24 W. Broniewskiego St., 71-460 Szczecin, Poland

**Keywords:** autosomal dominant polycystic kidney disease, gut microbiota, ketogenic diet

## Abstract

Polycystic kidney disease (PKD) includes autosomal dominant (ADPKD) and autosomal recessive (ARPKD) forms, both of which are primary genetic causes of kidney disease in adults and children. ADPKD is the most common hereditary kidney disease, with a prevalence of 329 cases per million in Europe. This condition accounts for 5–15% of end-stage chronic kidney disease (ESKD) cases, and in developed countries such as Poland, 8–10% of all dialysis patients have ESKD due to ADPKD. The disease is caused by mutations in the PKD1 and PKD2 genes, with PKD1 mutations responsible for 85% of cases, leading to a more aggressive disease course. Recent research suggests that ADPKD involves a metabolic defect contributing to cystic epithelial proliferation and cyst growth. **Aim:** This review explores the interplay between metabolism, obesity, and ADPKD, discussing dietary and pharmacological strategies that target these metabolic abnormalities to slow disease progression. **Conclusion:** Metabolic reprogramming therapies, including GLP-1 analogs and dual agonists of GIP/GLP-1 or glucagon/GLP-1 receptors, show promise, though further research is needed to understand their potential in ADPKD treatment fully.

## 1. Introduction

Polycystic kidney disease (PKD) encompasses both autosomal dominant polycystic kidney disease (ADPKD) and autosomal recessive polycystic kidney disease (ARPKD). Both entities represent the primary genetic causes of kidney disease in both adults and children [1]. The prevalence of autosomal dominant polycystic kidney disease (ADPKD) is approximately 1/400–1/1000 live births, with an estimated prevalence in Europe of 329 cases per million. These numbers make it the most common hereditary genetic kidney disease, accounting for nearly 5–15% of all cases of end-stage chronic kidney disease (ESKD) [2]. In most developed countries, including Poland, 8–10% of all dialysis patients have ESKD due to ADPKD [3]. The disease is caused by mutations in two genes, PKD1 (chromosome 16p13.3) and PKD2 (chromosome 4q21). Specifically, changes in PKD1 can be attributed to 85% of cases, while the majority of the remaining cases (10%) arise from changes in PKD2] [3,4]. PKD1 course is faster and more aggressive; patients with this variant require renal replacement therapy (RRT) approx. age of 45–55. PKD-2 course is less intensive; patients with this variant require RRT approx. age of 65 and more. One copy of the mutated gene from one parent is sufficient to develop the disease, and there is no clear gender or race-related predisposition. It is, therefore, important to diagnose and treat the disease at an early stage to improve patients’ quality of life and slow its progression. This systematic review examined recent evidence suggesting that a metabolic defect is likely linked to ADPKD, contributing to the proliferation of cystic epithelial cells and subsequent cyst growth. It also highlighted overlapping metabolic features and pathways between obesity and ADPKD. Furthermore, dietary and pharmacological strategies aimed at correcting metabolic abnormalities, considered potential treatments to slow ADPKD progression, were assessed.

### 1.1. Molecular Mechanism of the Disease

PKD1 and PKD2 code for two membrane proteins, polycystin-1 (PC1) and polycystin-2 (PC2), both associated with primary cilia. PC1 mediates cell–cell and cell–matrix interactions, acting as a receptor that regulates voltage-dependent calcium and potassium channels. Meanwhile, PC2 functions as a voltage-gated calcium channel. Together, these proteins form a complex on the primary cilia, playing a vital role in controlling intracellular calcium levels [5]. The interaction between PC1 and PC2 forms a complex on primary cilia responsible for the intracellular regulation of calcium levels [5]. PC1 is responsible for the mechanosensory function of primary cilia, cell adhesion and proliferation, and epithelial cell differentiation. Consequently, it is responsible for signaling affecting the processes of cellular growth and organization. PC2 plays a role in the regulation of calcium homeostasis in renal cells, specifically within the cell membrane and endoplasmic reticulum [6]. PC2 calcium channel dysfunction has been demonstrated to result in calcium homeostasis disorders, which in turn affect both apoptosis (programmed cell death) and cell proliferation. This, in turn, contributes to the development of cysts [7]. 

**Figure 1:**
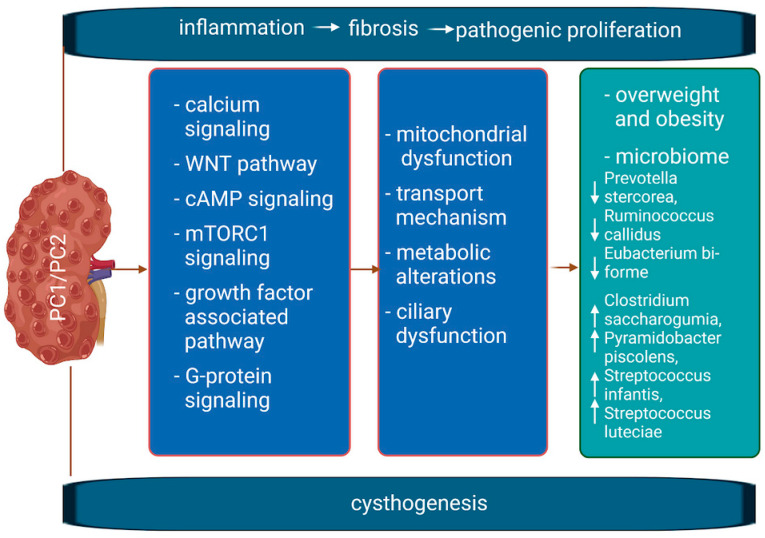
Generalized mechanisms involved in the development of PKD. Mutations in PKD1/PKD2 disrupt the normal function of various interconnected signaling pathways, leading to abnormal cell proliferation, fibrosis, and inflammation, all of which contribute to cyst formation. WNT pathway = a group of signal transduction pathways that begin with proteins that pass signals into a cell through cell surface receptors.

Figure 1 illustrates the interconnected pathological mechanisms underlying PKD, highlighting the fundamental concepts and the complex nature of the disease. Due to space constraints, we are unable to provide a comprehensive overview of the extensive PKD research. For a more in-depth exploration, readers are encouraged to consult the latest articles published by Dr. Albert Ong [2] and Dr. Jacob Torres [8].

Given the slow progression of the disease, various dietary strategies (including interventions in gut microbiome composition) and pharmacological approaches that may affect the progression of ADPKD should be considered. It is therefore recommended that treatment should include control of proteinuria, hypertension and elimination of smoking [9,10], adherence to a low-protein diet and dietary recommendations resulting from accompanying units, monitoring, and treatment of complications related to cysts and kidney stones, as well as treatment of urinary tract infections that may occur as a result of infection and dysbiosis [11]. Additionally, pain management should be considered [12,13].

### 1.2. Supplementation, Diet, Herbs, and Treatment in ADPKD

In the case of ADKPD patients, appropriate supplementation can support the health of patients, although it is not clear what changes in the microbiota composition result from kidney failure itself and which are due to medications, diet, and comorbidities. In one study, changes in the gut bacterial microbiota in patients with ADPKD at various stages of CKD were first presented [14]. Dietary interventions such as ketogenic diet, calorie restriction, and intermittent fasting are potential strategies for inducing metabolic reprogramming and slowing ADPKD progression [15]. It is worth noting that other studies have previously provided important results regarding dietary interventions in ADPKD. Specifically, Nowak [16] conducted a study comparing intermittent fasting and calorie restriction in obese patients with ADPKD. They found a correlation between slowed kidney growth and weight loss, regardless of the diet regimen [17]. This information is of great significance, as it highlights the necessity of maintaining a healthy body weight as the fundamental principle of dietary practice. A ketogenic diet, calorie restriction, intermittent fasting, and time-restricted feeding can reduce aerobic glycolysis and inhibit the mTOR pathway, resulting in decreased cyst cell proliferation, reduced kidney volume, and preservation of kidney function. mTOR signaling has been identified as a pivotal pathway regulating renal epithelial cells from the glomerular bundle to the terminal segment of the nephron. Both mTOR complexes, mTORC1 and mTORC2, have been demonstrated to regulate a number of processes, including glomerular filtration and the fine-tuning of tubular electrolyte balance. In the context of obstruction resulting from dysfunction of calcium and/or potassium channels, a reduction in cell proliferation may be a significant factor [18]. However, it is not a dietary element that directly affects the cause of the disease, so other modifications may contribute to achieving greater benefits for the patient. A balanced diet is recommended for individuals with ADPKD, with particular attention paid to maintaining optimal blood pressure, adequate hydration, moderate protein intake, and controlled levels of phosphorus and potassium. Additionally, the consumption of healthy fats, such as monounsaturated and polyunsaturated fats (MUFA, PUFA), and fiber is encouraged [10]. Increased fluid intake may be recommended during the early stages of the disease but should be avoided in patients with moderate to severe renal impairment. [19]. Regular consultation with a dietitian is essential for individuals with ADPKD to ensure their dietary needs and health status are met.

A key issue, especially in temperate and subarctic climates, is vitamin D supplementation, which should be monitored by a physician to avoid excessive calcium levels [20]. Vitamin D has renoprotective effects, potentially reducing blood pressure and inflammation. Moreover, low serum 25(OH)D levels and vitamin D receptor (VDR) expression are associated with larger kidney volume in patients with ADPKD [20]. Vitamin D deficiency is associated with proteinuria and higher mortality and may contribute to kidney dysfunction [21]. However, studies on vitamin D supplementation in this group of patients have not been conducted. The clinical benefits of supplementation with curcumin, ginkgolide B, saponins, vitamin E, or triptolide in patients with ADPKD remain uncertain. The combination of curcumin and ginkgolide B has been demonstrated to exert a synergistic inhibitory effect on cystogenesis. The anti-cystogenic effects of curcumin were found to be mediated by the blockade of the epidermal growth factor receptor (EGFR)/extracellular signal-regulated kinases 1 and 2 (ERK1/2), c-Jun *N*-terminal kinases (JNK), and phosphatidylinositol 3-kinase (PI3K)/mammalian target of rapamycin (mTOR) signaling pathways. In contrast, ginkgolide B was observed to inhibit cystogenesis by reducing the expression of EGFR/ERK1/2, JNK, and p38 signaling pathways [22]. Conversely, curcumin supplementation did not enhance blood vessel function or decelerate kidney growth in pediatric and young adult patients with ADPKD [23]. One study indicates Saikosaponin-d (SSd), an inhibitor of the sarcoplasmic/endoplasmic reticulum Ca2+ ATPase (SERCA) pump, as a possible therapeutic target [24]. Notably, β-hydroxybutyrate supplements have shown promise in animal models; however, their safety and efficacy in ADPKD require further evaluation through well-designed clinical trials [25]. In addition to single-treatment studies targeting metabolic reprogramming in ADPKD, there is strong potential to combine these therapies with compounds that target other altered signaling pathways, such as vasopressin receptor antagonists. Pharmacological treatment trials also include the use of AMP-activated kinase activators (AMPK), sodium-glucose cotransporter-2 inhibitors (SGLT-2i), niacinamide, thiazolidinediones, GLP-1 agonists, and mTOR inhibitors (mammalian target of rapamycin). AMPK is highly expressed in the kidney and regulates a number of physiological processes. It participates in carbohydrate and lipid metabolism, regulation of cell cycle and mitochondrial energetics, and inhibits inflammation and fibrosis [26]. Renal cystic tissues exhibit a metabolic reprogramming profile that mimics the “Warburg effect” of proliferative tissues (aerobic glycolysis) [26]. Furthermore, AMPK regulates cell growth by inhibiting the serine-threonine protein kinase mTORC. Song et al. suggest that metformin is an indirect activator of AMPK with a very good safety profile—both features making it an attractive candidate for the treatment of ADPKD [27]. Salsalate was also effective in ameliorating the severity of cystic disease by reducing the incidence of renal failure (BUN > 20 mmol/l), KW/BW. Salsalate treatment was associated with reduced mTORC1 activity and cell proliferation [27].

The effects of herbs and natural supplements on ADPKD patients can be unpredictable, particularly in cases where they affect kidney function. It is imperative that patients consult with a qualified medical professional prior to initiating any herbal or natural supplement regimen. There is a paucity of studies in the scientific literature on this topic. A number of studies have been conducted on this topic, but none have included a follow-up phase [28]. It appears that herbs with diuretic properties may assist in the elimination of excess fluids and toxins from the body. Such herbs include nettle, dandelion, parsley, and cranberries, which possess antibacterial properties. It is not advisable to consume licorice, as it has been linked to sodium and water retention, which can contribute to the development of hypertension [29]. Furthermore, fenugreek is contraindicated because of its potential to elevate potassium levels, particularly in individuals with the PKD1 mutation. However, the excessive intake of silica can result in an increased burden on the kidneys and disturbances in electrolyte balance [30]. It is therefore recommended that herbs containing significant amounts of silica, such as horsetail, bamboo, green oats, and, for the same reasons, nettle and dandelion, should be used with caution. At present, no studies have been conducted in this area. However, this is the opinion recommended by the authors of this review.

To determine the quality and quantity of physical activity that patients can safely perform, it is necessary to thoroughly assess the multisystemic nature of the disease, especially the involvement of the cardiovascular system [25].

## 2. Materials and Methods

**Figure 2:**
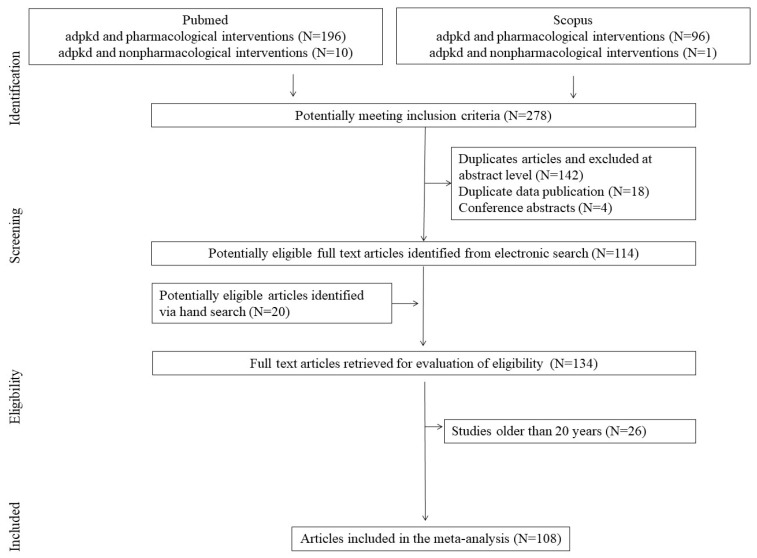
Research search flowchart.

The present review evaluates the above-mentioned topics considering the literature published up to 31 July 2024. A systematic literature search has been conducted based on the PubMed (N = 206) and Scopus (N = 97) databases. The passwords were checked for combined terms: ADPKD and pharmacological interventions, ADPKD and nonpharmacological interventions. These terms were combined with nutrition, supplementation, diet, probiotics, microbiota, and inflammation. Studies that were not in the English language, letters to the editor, and abstracts to conferences were excluded, as shown in the flow chart (Figure 2). Finally, studies older than 20 years were excluded from the database. All included studies were screened and discussed by a minimum of three authors until a general consensus was reached.

## 3. ADPKD, a Disease with Dysregulated Metabolism: In Vitro and Animal Model Studies

In ADPKD, changes in the cellular metabolism of the kidneys are observed. To better understand the role of these changes in the pathogenesis of ADPKD, it is proposed that future research directions focus on the issue of dysregulated metabolism and factors altering signaling cascades, as in the case of tolvaptan [16]. Numerous studies in recent years have confirmed that dysregulated metabolism, e.g., glucose metabolism, plays a key role in the development of ADPKD (Table 1). 

## 4. Metabolic Reprogramming: A Common Feature in Human ADPKD

Currently, the dysregulation of cellular metabolism in the kidneys of ADPKD patients has only been partially confirmed. The HALT Progression of Polycystic Kidney Disease (HALT-PKD A and B) study suggested a link between two long-chain triglycerides and total kidney volume (TKV) [46]. Changes in fatty acid metabolism, including lipoxygenase pathways, have been observed [47]. Compared with other CKDs, including glomerular diseases, detectable metabolic changes in ADPKD appear unique and specific [46]. Consistent with the hypothesis of impaired fatty acid metabolism and mitochondrial β-oxidation is the increase in 16-hydroxyplamatate levels [48]. There is indirect evidence that ADPKD patients suffer from metabolic impairments. Lower HDL cholesterol is associated with kidney enlargement, while increased uric acid excretion and low urinary citrate levels are common, especially in those with kidney stones [49]. Insulin resistance or impaired secretion may also play a role in ADPKD, though these data are inconsistent [50]. Patients with both type 2 diabetes and ADPKD have notably larger kidneys than those without diabetes, indicating that insulin resistance could contribute to disease progression. Furthermore, there is a notable association between ADPKD and a heightened risk of developing diabetes post-kidney transplant [51].

In summary, the current studies not only strongly suggest that metabolic alterations are important in the pathogenesis of PKD but also highlight the potential of various therapeutic strategies to control it.

### 4.1. Metabolic Pathway Changes in Overweight and Obesity: Parallel Metabolic Disturbances in ADPKD

Patients with ADPKD tend to experience an increase in body mass index (BMI) over time, mirroring trends in the general population [52]. About two-thirds of adults with ADPKD are classified as overweight or obese, which is a recognized risk factor for chronic kidney disease and end-stage renal disease. Weight loss has been shown to slow the decline in kidney function [53,54,55]. Recent research, such as the HALT-PKD study (in which 441 patients were observed for 5 years), has revealed that being overweight or obese significantly accelerates kidney growth in ADPKD, with obese individuals experiencing a faster decline in kidney function [53]. Excessive food intake and obesity activate the mTOR (mechanistic target of rapamycin) pathway through the PI3K/Akt and IGF-I signaling pathways, along with AMPK [56]. Notably, overstimulation of mTOR has been linked to the onset of diabetes [57]. Reduced AMPK activity observed in obese rodent models suggests that AMPK activation could offer a promising therapeutic strategy for PKD [58]. This points to a potential connection between higher body mass index (BMI) and PKD, indicating that increased BMI might contribute to the progression of PKD or that PKD itself could promote obesity.

### 4.2. Dietary Strategies to Address Metabolic Abnormalities

Several dietary approaches have shown promise in slowing ADPKD progression. These include daily calorie restriction, intermittent fasting (fasting or significantly reducing caloric intake for 1–3 days per week), time-restricted feeding (limiting food intake to a specific window each day), and altering macronutrient composition, such as adopting low-fat, low-protein, or low-carbohydrate diets.

### 4.3. Nutritional Approaches Using Non-PKD Rodent Models

Dietary interventions aimed at metabolic pathways have been extensively explored in rodent models without PKD. Sustained calorie restriction, combined with sufficient nutrition, has been shown to prolong lifespan in many animal studies and improve key health metrics related to aging, such as body weight, lipid levels, and glucose regulation [59]. Additionally, calorie restriction has been found to offer protection against ischemia-reperfusion kidney injury in rodents, likely through mechanisms such as enhanced autophagy and greater stress resistance or preconditioning [60]. 

Intermittent fasting has also yielded several positive effects in non-PKD rodent models, including lower fasting glucose and insulin levels, better insulin sensitivity, improved lipid profiles, and reductions in both blood pressure and heart rate. It has been shown to mobilize glycogen and fat stores, leading to increased production of ketone bodies and gluconeogenesis, which could result in a shift from carbohydrate to fat metabolism [61].

In a prominent study on Drosophila [62], time-restricted feeding was shown to slow the aging of the heart, improve sleep quality, and prevent weight gain, all without changes to total calorie intake or activity levels. Similarly, in rodents, time-restricted feeding helped prevent weight gain from high-fat, high-sucrose diets and improved overall body composition [63,64]. This approach also enhanced insulin sensitivity, similar to intermittent fasting. In mice fed a high-fat diet, time-restricted feeding improved mTOR and AMPK signaling when compared with those fed without restriction [64].

### 4.4. Calorie Restriction and Fasting Trials in Humans without ADPKD

Most clinical trials on calorie restriction have been conducted on healthy populations, focusing on weight loss and insulin sensitivity rather than disease-specific outcomes. However, the CALERIE trial, which involved 220 healthy volunteers over two years, demonstrated that a 25% reduction in daily caloric intake was feasible, safe, and led to improvements in reductions in inflammatory markers, cardiometabolic risk factors, despite some concerns about bone loss and anemia [65].

Research on intermittent fasting in healthy, overweight, and obese adults has demonstrated that it is both practical and safe [66,67,68]. Beyond aiding in weight and fat reduction, intermittent fasting has been linked to decreased oxidative stress, lower inflammation, reduced blood pressure, and improved lipid levels.

A newer approach, time-restricted feeding (TRF), offers an alternative method for weight management, particularly for those with a normal BMI, and may be more sustainable than intermittent fasting. TRF leverages similar evolutionary mechanisms, illustrating how the timing of meals can significantly impact metabolic processes [69]. Although randomized controlled trials (RCTs) on TRF are fewer compared with intermittent fasting, one 16-week pilot study in overweight individuals found that reducing daily caloric intake by 20% led to moderate weight loss, improved energy levels, and better sleep, with these effects lasting up to a year [69].

### 4.5. Calorie Restriction and Nutrient Availability in ADPKD

Moderate daily calorie restriction (ranging from 10–40%) has been shown to significantly slow kidney enlargement and enhance kidney function in animal models of ADPKD [33,70]. This effect is partly due to the suppression of mTOR signaling, activation of AMPK, and a reduction in IGF-I levels [70]. Moreover, calorie restriction reverses the ADPKD-related increase in hexokinase 2 expression, a key enzyme in glycolysis, promoting metabolic reprogramming [33]. Improvements in kidney fibrosis, inflammation, and damage markers have also been observed in a dose-dependent manner. Notably, a 40% calorie reduction, even when initiated in the later stages of the disease, not only slows ADPKD progression but can also reverse it [33]. Time-restricted feeding has also shown promising results in the Han:SPRD rodent model of PKD [71]. When rodents were allowed ad libitum feeding within an 8-h window during their active phase, significant reductions in cyst formation, kidney fibrosis, and improved kidney function were observed, along with the normalization of mTORC1 and STAT3 signaling—all without altering total calorie intake or body weight. Similar benefits were seen with a ketogenic diet or supplementation with the natural ketone β-hydroxybutyrate, which promotes metabolic reprogramming by shifting fuel sources, lowering glucose availability, and increasing fatty acid oxidation [8].

Additionally, protein restriction has been beneficial in improving PKD outcomes in two rodent models [72], while a soy-based diet has been shown to halt PKD progression by modifying lipid metabolism [73]. Reducing lipid intake has also been found to impact cyst growth in these models [31].

## 5. Pharmacological Alternatives to Diet Changes Modulating Metabolic Disorders in ADPKD

The practical application of dietary modifications is limited. Nutritional changes can be challenging to implement and maintain over the long term. In light of this, pharmacological treatments targeting metabolic reprogramming may be a sought-after alternative in slowing ADPKD progression.

### 5.1. Tolvaptan

Tolvaptan, a highly selective V2 vasopressin receptor (AVP) antagonist, is the only approved therapy specifically for treating ADPKD. Research demonstrates that Tolvaptan slows the progression of cyst growth and curbs the increase in total kidney volume (TKV), effectively delaying the onset of end-stage kidney disease (ESKD). Normally, AVP binds to V2 receptors in the distal nephron and collecting ducts, stimulating the production of cAMP, which drives cyst formation. Tolvaptan works by blocking this AVP binding, leading to aquaresis (water excretion without sodium loss), reduced urinary osmolality, and a decrease in cyst proliferation by inhibiting cAMP production. Currently, Tolvaptan is recommended for ADPKD patients who are at high risk of rapid disease progression [74].

Nowadays, therapy with tolvaptan should be recommended to all ADPKD patients with probable rapid progression of the disease. Criteria for tolvaptan use are proposed as follows: 1. age 18–55 years, 2. chronic kidney disease CKD 1–4 (eGFR ≥ 25 mL/min per 1.73 m^2^), 3. high risk measured by available risk scores (longitudinal diameter > 17 cm by ultrasound, total kidney volume > 750 mL, Mayo imaging classification 1C, 1D, 1E or PROPKD score > 6), and 4. rapid decline of eGFR 3 mL/min per > 1.73 m^2^) for 5 years. 

The Mayo imaging classification, developed from MRI data, is a reliable predictor of eGFR decline based on initial kidney volume [75]. Similarly, the PROPKD score estimates disease prognosis by integrating genetic information with clinical factors such as sex, age at onset of hypertension, age at first occurrence of macroscopic hematuria, and cyst-related flank pain [76]. Risk stratification is crucial, as individuals with a low likelihood of ADPKD progression tend to experience no significant decline in eGFR [77]. Therefore, Tolvaptan is not recommended for patients with ADPKD who are anticipated to have a mild disease trajectory.

### 5.2. AMPK Activators

Metformin, an FDA-approved activator of the AMPK pathway, is widely used for treating type 2 diabetes and polycystic ovary syndrome. Research involving kidney epithelial cell cultures and PKD mouse models has shown that metformin inhibits mTOR signaling and the cystic fibrosis transmembrane conductance regulator (CFTR), reducing epithelial proliferation and secretion, thereby slowing cyst growth [78,79]. Similarly, other studies on animal models have demonstrated that statins, also AMPK activators, can minimize cyst formation, preserve kidney function, and reduce gut inflammation and fibrosis [80]. Currently, two prospective randomized controlled trials (RCTs) are underway to investigate metformin as a potential treatment for ADPKD. The first, titled “Trial for Administration of Metformin to Tame PKD” (NCT0256017), is a phase II, double-blind, placebo-controlled RCT. It spans 26 months and includes 96 non-diabetic adults aged 18–60 with ADPKD and eGFR greater than 50 mL/min per 1.73 m^2^. The study’s primary objectives are to evaluate the safety and tolerability of metformin, while secondary measures focus on changes in total kidney volume (TKV), liver volume, pain, quality of life, and biomarkers related to the glycolytic and AMPK pathways. The second RCT (NCT02903511) involves 50 non-diabetic adults aged 30–60 years with ADPKD and an eGFR of 50–80 mL/min per 1.73 m^2^. This year-long study primarily evaluates the proportion of participants who are able to take the full or at least 50% of the prescribed dose after 12 months. Secondary endpoints include changes in TKV and eGFR. Additionally, a direct comparison trial between metformin and tolvaptan is planned in Italy (NCT03764605) for 2020, involving 150 adults aged 18–50 years with eGFR ≥ 45 mL/min per 1.73 m^2^ and a PKD1 mutation. However, this study has yet to commence. Statins, another AMPK activator approved by the FDA, have been shown to slow kidney growth in a three-year RCT involving children and young adults with ADPKD [81]. An ongoing RCT in which 150 pts, 40 mg of pravastatin, or placebo are participating is evaluating whether a 2-year pravastatin treatment provides similar benefits in TKV change in adults with ADPKD and preserved kidney function (NCT03273413).

### 5.3. Sodium-Glucose Cotransporter-2 Inhibitors

Sodium-glucose cotransporter-2 (SGLT2) inhibitors are relatively newer drugs that lower blood sugar by increasing glucose excretion in urine. In a rat model (Han:SPRD) of polycystic kidney disease (PKD), phlorizin, an SGLT1/2 inhibitor, reduced cyst formation after five weeks of treatment [82]. However, the SGLT2 inhibitor dapagliflozin improved kidney function and reduced albuminuria but did not stop cyst growth [83] and even increased cyst size in a different rat model of autosomal dominant polycystic kidney disease (ADPKD) [84]. Similarly, canagliflozin showed no benefit in reducing kidney disease severity in an ADPKD model [85]. Yet, in a study of 4401 patients with type 2 diabetes and chronic kidney disease (CKD), canagliflozin (100 mg) lowered the risk of kidney failure and cardiovascular events, prompting early trial termination [86].

Caution is recommended when considering SGLT2 inhibitors for ADPKD treatment.

### 5.4. Niacinamide/Nicotinamide

Niacinamide (nicotinamide), a form of niacin, inhibits the NAD-dependent enzyme SIRT-1, which acts as a metabolic sensor. SIRT-1 expression is elevated in the kidneys of several polycystic kidney disease (PKD) rodent models [84]. Niacinamide has been shown to slow cyst growth and improve kidney function in two mouse models of autosomal dominant polycystic kidney disease (ADPKD), likely through SIRT-1 inhibition [87]. Interestingly, while calorie restriction activates SIRT-1 [59], it doesn’t seem to affect SIRT-1 levels in PKD kidneys [33]. A phase II trial (NCT02558595) is evaluating niacinamide in ADPKD patients over one year. Early trials (N = 10 and N = 36) tested niacinamide’s biological activity and safety, with primary outcomes focused on changes in SIRT-1-mediated p53 acetylation. Other measures include adjusted total kidney volume, eGFR, and monocyte chemoattractant protein-1.

### 5.5. Thiazolidinediones

Thiazolidinediones, FDA-approved drugs for managing high blood glucose in type 2 diabetes, activate PPARγ, a regulator of fatty acid and glucose metabolism. They also inhibit cystic fibrosis transmembrane conductance regulator activity and chloride secretion in response to vasopressin [88]. Pioglitazone and rosiglitazone have been effective in reducing cyst formation in several animal models of polycystic kidney disease (PKD) [89,90]. A 1-year phase II crossover study is currently evaluating the safety and efficacy of pioglitazone in non-diabetic adults with autosomal dominant polycystic kidney disease (ADPKD), with safety as the primary focus and total kidney volume (TKV) as a secondary endpoint (NCT02697617). However, the FDA has issued a warning about a potential increased risk of bladder cancer with pioglitazone, urging caution.

### 5.6. Analogues and Agonists of Gut Hormones

GLP-1 analogs, which have an established role in the treatment of type 2 diabetes and obesity, can also be used in the treatment of individuals with liver and kidney complications [91,92,93]. One of the appealing aspects of the new generation of GLP-1 analogs is the significant weight reduction (>10%) achieved in obese and diabetic patients [94]. Importantly, GLP-1 exerts its effects by binding to the GLP-1R receptor and activating adenylate cyclase, leading to an increase in cAMP production. Therefore, it is necessary to investigate the expression of GLP-1R in cyst epithelium and the potential impact of GLP-1 agonism on cAMP levels [91,95].

#### 5.6.1. Dual Agonists of GLP-1 and Glucagon Receptors

Dual agonists for the GLP-1 and glucagon receptors show a stronger weight-reducing effect compared with selective GLP-1 agonism [96,97]. Glucagon reduces mTORC1 levels and stimulates AMPK [98] and ketogenesis [99,100]. Therefore, the simultaneous agonism of GLP-1R and glucagon receptors represents a potential therapeutic approach for polycystic kidney disease (ADPKD). However, a recent observational study involving 664 ADPKD patients from the HALT study database, who exhibited elevated endogenous glucagon levels, did not demonstrate any protective effect of glucagon in the context of ADPKD [101]. Further research is needed to determine the relationship between glucagon and ADPKD, as well as to assess receptor expression in kidney cysts. Future studies will clarify the potential effectiveness of GLP-1 agonists in reducing body weight and their other beneficial effects in the context of ADPKD.

#### 5.6.2. Dual Agonist for GLP-1 and GIP Receptors (Tirzepatide)

No specific trial is available or ongoing exploring the impact of tirzepatide in patients with CKD. However, a post hoc analysis of pre-specified kidney endpoints was reported for SURPASS-4 [102] that compared tirzepatide and insulin glargine. Tirzepatide participants presented significantly fewer composite kidney endpoints (eGFR decline ≥ 40% from baseline, renal death, kidney failure, or new-onset macroalbuminuria, i.e., UACR > 300 mg/g, A3) [HR 0.59 (95% CI 0.43–0.80); *p* < 0.05]. Tirzepatide kidney protection was mainly explained by a reduction in the new onset of macroalbuminuria [HR 0.41 (95% CI 0.26–0.66); *p* < 0.05] in the full population and patients with A2 albuminuria. Interestingly, the decrease in new-onset macroalbuminuria) was apparent in both patients on and off SGLT2 inhibitors, although the difference was only statistically significant for patients off SGLT2 inhibitors.

### 5.7. m TOR Inhibitors

In humans with ADPKD, the mTOR inhibitors rapamycin and everolimus have demonstrated no clear-cut benefit. After 2 years of everolimus/placebo treatment in 433 patients (2 × 2.5 mg or placebo), increasing TKV slowed transiently but renal function worsened [103]; 18 months of rapamycin therapy in 100 patients receiving rapamycin (2 mg daily) or standard of care had no effect on TKV but was associated with a trend toward slowing of renal function decline [104]; and 6 months of rapamycin treatment had no protective effect on renal function or TKV but was associated with a decrease in the relative change and a trend toward an absolute change in renal cystic volume [105]. Currently, it appears that research on the use of mTOR in ADPKD, despite a solid theoretical foundation and a proven mechanism of action, has not demonstrated sufficient effectiveness in limiting cyst growth and will not be continued because of the emergence of better therapeutic options.

## 6. Polycystic Kidney Disease and Microbiota

This is an area where minimal research has been conducted. In patients with advanced kidney disease and/or undergoing dialysis, the gut microbiota undergoes changes. However, it is not clear what changes in the microbiota composition result from kidney failure itself and which are due to medications, diet, and comorbidities [14]. A direct impact of reduced kidney function on microbiome composition was described using rigorous criteria to eliminate the possibility of other confounding factors and analyze the direct impact of the uremic environment. The microbiome was analyzed using three different approaches to more accurately assess the impact of mild, moderate, and advanced kidney failure on the microbiome [14]. Stool samples from ADPKD patients at various CKD stages were examined: dialysis, eGFR 15–60, and >60 mL/min/1.73 m^2^. Participants were enrolled to achieve an equal distribution across groups, targeting a final ratio of 1:1:1. Out of 1962 identified patients, strict inclusion and exclusion criteria were applied. To maintain the desired ratio, PKD patients with CKD stage V-ESRD were recruited first, followed by the remaining groups. Patients with eGFR < 45 mL/min showed a significantly lower relative abundance of the bacteria phylum Tenericutes. Species and genus-level analysis revealed statistically significant relative abundances of fourteen species and nine genera. The relative abundance of Lactobacillus iners was increased in patients with eGFR < 45 mL/min, while the relative expression of Prevotella stercorea, Ruminococcus callidus, and Eubacterium biforme was decreased [14]. Patients on HD expressed lower α diversity compared with the rest. The relative abundance of 21 species and 18 genera was differentially expressed between the two groups. Clostridium saccharogumia, Pyramidobacter piscolens, Streptococcus infantis, and Streptococcus luteciae relative abundances were increased in HD patients, while Ruminococcus callidus expression was decreased [14].

In another available study [106], the microbiome of infected cysts, stool, and saliva in ADPKD patients was examined. It is believed that bacteria causing cyst infection originate from the patient’s gut translocation rather than blood, as blood cultures in such patients are often negative. The researchers presented descriptions of two ADPKD patient cases with kidney cyst infections. In both cases, the microbiome composition in cysts was completely different from stool or saliva. Additionally, the proportion of major genera and phyla in cyst fluid was much lower than in stool and saliva. The finding that causative bacteria were not widely distributed in stool and saliva suggests that bacterial mass may not be significant for bacterial translocation. The authors suspect that it is related to the ability of each bacterial species to invade and migrate through the intestinal mucosal barrier. Indeed, the most frequently migrating bacteria are aerobic pathobionts, including Enterococcus, Enterobacteriaceae (e.g., E. coli and Klebsiella spp.), and viridans streptococci [106]. Previous studies have shown that prior exposure to antibiotics affects the gut microbiome. Specifically, cephalosporins and quinolones decreased E. coli abundance, and cephalosporins and carbapenems increased Enterococcus spp. abundance and carbapenems and quinolones strongly decreased anaerobic bacteria abundance [107].

The limited number of studies on the gut microbiome in ADPKD patients does not allow for conclusive conclusions and indicates the need for further research, which is highly needed in the context of slowing ADPKD progression and effectively treating complicated cyst infections.

## 7. Recommendations

Our review sheds new light on the role of diet, gut microbiota, supplementation, and treatment in the complex disease that is ADPKD. It appears that maintaining a healthy body weight is an essential element of diet. It is known that excessive food intake and obesity activate mTOR through PI3K/Akt, IGF-1, and AMPK.

**Figure 3:**
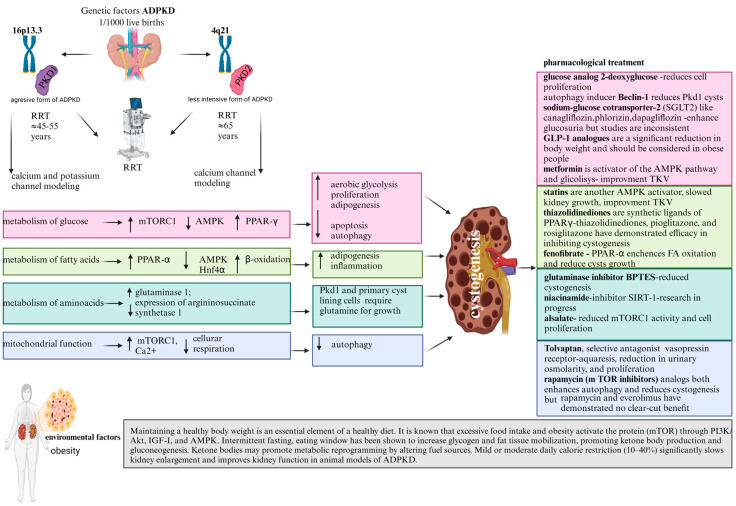
Current state of knowledge of pharmacological and dietary metabolic modifications in ADPKD. ADPKD—autosomal dominant polycystic kidney disease; PKD—polycystic kidney disease; RRT—renal replacement therapy; mTORC—mammalian target of rapamycin complex; AMPK—activated protein kinase; PPAR—peroxisome proliferator-activated receptor; GLP—glucagon-like peptide; TKV—total kidney volume; FA—fatty acids; SIRT—silent information regulator). BPTS Bis-2-(5-phenylacetamido-1,3,4-thiadiazol-2-yl)ethyl sulfide.

Limiting the eating window has been shown to boost glycogen and fat tissue mobilization, leading to increased ketone body production and gluconeogenesis. These ketone bodies may aid in metabolic reprogramming by altering energy sources. In ADPKD animal models, mild to moderate calorie restriction (10–40%) has been shown to reduce kidney enlargement and improve kidney function significantly. Medications are an integral part of combination therapy. The recommended dose and form of vitamin D3 depend on the stage of kidney disease. At stages 3b-5 of CKD, the active form of vitamin D3, 1–25 OH D3 (alfacalcidol, calcitriol), is recommended. At earlier stages of CKD, cholecalciferol may be used. The recommended doses of metformin range from 1000–3000 mg/24 h, depending on eGFR. The dose of tolvaptan is individualized, typically between 60–120 mg per day in divided doses, depending on treatment tolerance and liver function tests, with the goal of reaching the highest tolerated dose. A therapeutic option to consider in combination therapy includes statins (atorvastatin and pravastatin do not require dose adjustment based on eGFR) and fenofibrate 145–300 mg/day, adjusted for eGFR, which may slow cyst growth due to their mechanism of action. Niacin at a dose of 400–1000 mg may also be used as a supportive treatment in combination therapy. Rosiglitazone is not available in Europe, while pioglitazone at a dose of 15–45 mg/day should be used with caution in patients with congestive heart failure because of the risk of edema. The current state of knowledge regarding pharmacological and dietary metabolic modifications is presented in the figure below (Figure 3).

## 8. Conclusions, Limitations, and Future Directions

- ADPKD is linked to metabolic defects that contribute to cyst growth, with overlaps seen between ADPKD, obesity, and related conditions. Dietary and pharmacological strategies targeting these issues are explored as potential therapies.
- Metabolic reprogramming therapies show promise in slowing ADPKD progression, but more research is needed, especially for treatments such as GLP-1 analogs and dual agonists that target specific pathways.
- Dietary interventions have limitations, such as long-term adherence challenges, and weight loss is not suitable for everyone, requiring careful nutrition management.
- Combining metabolic reprogramming therapies with drugs such as tolvaptan could enhance treatment, reduce side effects, and minimize dietary restrictions, but further research is needed, especially regarding use in children.

## Figures and Tables

**Table 1:** In Vitro and Animal Model Studies showing Dysregulated Metabolism in PKD.

Mechanism	Description	Model	Author/Year
Dysregulated glucose metabolism	Studies on embryonic fibroblasts from Pkd1−/−mice have shown that altered, reprogrammed cells favor aerobic glycolysis (Warburg effect), increase the mammalian target of rapamycin complex 1 (mTORC1) levels, inhibit AMPK activation, increase proliferation, decrease apoptosis, and cause defective autophagy. Changes in glucose metabolism have been described using in vivo models evidenced by increased expression of key glycolytic genes in the kidneys of patients with cystic epithelium and kidneys of mice with polycystic kidney disease (PKD)	Mouse embryonic fibroblasts	[26] (Rowe et al., 2013) [31] (Menezes et al., 2016)
Dysregulated glucose metabolism	Blocking glycolysis with 2-deoxyglucose, a glucose analog that cannot be metabolized, has been shown to reduce cell proliferation in human PKD cells and slow kidney cyst formation in mice. Based on these studies, higher glucose concentrations increase kidney cyst growth, promote cystogenesis, and cause structural and functional kidney damage in the rodent PKD model.	Mouse kidneys (N = 6) Mice (N = 25) Mouse embryonic fibroblasts Mouse kidneys (N = 12) Mice (N = 15)	[32] (Chiaravalli et al., 2016) [33] (Warner et al., 2016) [34] (Kraus et al., 2016) [31] (Menezes et al., 2016) [26] (Rowe et al., 2013) [35] (Sas et al., 2015)
Altered lipid metabolism and reduced fatty acid oxidation	This mechanism is believed to involve hepatocyte nuclear factor 4α (Hnf4α) or peroxisome proliferator-activated receptor α (PPARα). In fact, using the PPARα agonist fenofibrate in ADPKD models has been found to enhance fatty acid oxidation and alleviate cyst formation. Conversely, the absence of Hnf4α in PKD models worsens the severity of the cystic disease.	Mouse kidneys (N = 10) human cyst lining cells Mice (N = 25)	[36] (Lakhia et al., 2018); [37] Soomro et al., 2018) [33] Warner et al., 2016
Altered amino acid metabolism	The level of glutaminase 1 in the epithelium lining cysts in human ADPKD kidneys and mice models is increased. Increased levels of glutaminase 1 have been observed in the epithelial lining of cysts in both human ADPKD kidneys and mouse models. Glutamine is essential for the growth of both Pkd1 mutant cells and ADPKD cyst-lining cells, suggesting a dependence on glutamine in PKD. Therefore, inhibiting glutamine metabolism with glutaminase inhibitors such as BPTES or CB839 reduces cyst formation in specific PKD models. However, CB839 proved ineffective in other models, suggesting that cyst growth may also rely on arginine, as argininosuccinate synthetase 1 expression is reduced in ADPKD, and arginine deficiency triggers its upregulation, reducing cystogenesis.	Mouse embryonic kidneys (N = 20) Mouse tubular cell lines	[38] Flowers et al., 2018; [39] Trott et al., 2018
Defects in autophagy and mitochondrial function	Autophagy, a process critical for maintaining cellular energy balance, is typically activated in response to nutrient deprivation. In PKD cells, however, this process is disrupted, primarily due to impaired fusion between autophagosomes and lysosomes, a phenomenon known as defective autophagic flux. Reduced expression of the autophagy-related protein Atg5 has been linked to enhanced cyst formation, while activating autophagy with Beclin-1 has been shown to decrease cyst development in Pkd1 models. Interestingly, while trehalose, a natural autophagy stimulant, failed to mitigate Pkd1 disease, targeting mTORC1, a key autophagy suppressor, with a rapamycin derivative led to both improved autophagy and decreased cyst growth in PKD.	Mice (N = 41) Zebrafish cell lines Han:SPRD rats (N = 6) Cpk mice (N = 7) Pkd2WS25/−mice (N = 4)	[40] Chou et al., 2019; [41] Zhu et al., 2017; [42] Belibi et al., 2011;
Mitochondrial dysfunction	Mitochondrial dysfunction in ADPKD is partly due to abnormalities in their structure and biogenesis. Kidney tissue from ADPKD patients and mouse models shows fragmented, swollen mitochondria with irregular movement and reduced mitochondrial DNA. Additionally, mitochondrial function is compromised, with increased reactive oxygen species production, elevated Ca2+ uptake, and diminished cellular respiration. Notably, both polycystin-1 and-2 have been found to influence mitochondrial function directly.	Cell culture Pkd1 human knockout cell lines Human embryonic kidney cells	[43] Kuo et al., 2019; [44] Lin et al., 2018; [45] Padovano et al., 2017

## Data Availability

Not applicable.

## Abbreviations

ADPKD—autosomal dominant polycystic kidney disease; PKD—polycystic kidney disease; RRT—renal replacement therapy; mTORC—mammalian target of rapamycin complex; AMPK—activated protein kinase; PPAR—peroxisome proliferator-activated receptor; GLP—glucagon-like peptide; TKV—total kidney volume; FA—fatty acids; SIRT—silent information regulator.
